# Chronic neck pain is associated with increased mortality in individuals with osteoarthritis: results from the NHANES database prospective cohort study

**DOI:** 10.1186/s13075-023-03103-w

**Published:** 2023-07-19

**Authors:** Xi Chen, Lihua Gong, Cheng Li, Siyuan Wang, Yixin Zhou

**Affiliations:** 1 Department of Adult Joint Reconstructive Surgery, Peking University Fourth School of Clinical Medicine, 31 East Xinjiekou Street, Beijing, 100035 China; 2 Department of Pathology, National Cancer Center/National Clinical Research Center for Cancer/Cancer Hospital, Chinese Academy of Medical Sciences and Peking Union Medical College, Beijing, 100021 China

**Keywords:** Osteoarthritis, NHANES, Risk factor, Neck pain, Mortality

## Abstract

**Background:**

Neck pain (NP) is a common symptom reported in the elderly. However, no study has examined the relationship between NP and osteoarthritis (OA) so far, and this study aimed to investigate the association of neck pain with the prevalence and mortality of OA.

**Methods:**

A total of 5965 participants were included in this cohort study based on the National Health and Nutrition Examination Survey data set of the USA (NHANES). Death outcomes follow-up information was ascertained by linkage to National Death Index (NDI). The association between NP and OA was studied by multi-various logistic regression models after adjusting for potential confounding factors. Cox proportional hazards models were used to elucidate the relationship between NP and all-cause mortality in OA patients.

**Results:**

Among all participants, 8.18% had osteoarthritis, and 5.92% suffered from neck pain. Neck pain was associated with osteoarthritis [1.932 (1.232, 3.028), *p* < 0.01], which still reminded significant after adjustments [2.519 (1.325, 4.788), *p* < 0.01] and stratified analysis by sex, race, and smoke status. In OA patients, chronic neck pain (over 1 year) was significantly associated with higher risks of all-cause mortality before [2.94 (1.61, 5.37), *p* < 0.01] and after adjustment [3.30 (1.23, 45.85), *p* < 0.05].

**Conclusion:**

Neck pain was strongly associated with osteoarthritis. Moreover, chronic neck pain over 1 year significantly increased the mortality of OA patients. Our study demonstrates the need to screen osteoarthritis in the neck pain population and select a more appropriate treatment strategy promptly for those patients.

## Introduction

Osteoarthritis (OA) is a major public health concern worldwide, which has affected over 500 million people and increased by 113.25% over the past decades [1]. As a chronic disease, it has become a severe clinical and public health problem for patients and clinical practitioners with rapid growth in medical and nursing care costs. A plethora of studies has revealed many risk factors of OA including age, female gender, higher BMI, higher BMD, smoking status, and genetic factor [2–13].

However, there are few studies focus on the relationship between spinal factors and OA in the elderly. Furthermore, to our knowledge, there is no previous study that investigated the association between neck pain (NP) and OA, and how it affected the mortality of OA patients. Several systematic reviews unveiled that the mortality of OA varied with different comorbidities and different chronic conditions [14–16]. Neck pain is one of the comorbidities in OA patients reported by previous studies [14, 17]. It is important to uncover the association of neck pain and osteoarthritis for clinical practice and the development of clinical guidelines.

Therefore, the primary aim of our study was to explore the relationship between neck pain and osteoarthritis based on the data from the National Health and Nutrition Examination Survey data set of the USA (NHANES). The secondary objective was to determine the association of neck pain and all-cause mortality in patients with OA. Our hypothesis is that individuals with chronic neck pain may experience an impact on the mortality rate associated with osteoarthritis (OA). This is because neck pain has the potential to limit mobility and physical activity in patients with OA, which could lead to a sedentary lifestyle and a decrease in overall physical fitness [18, 19]. We hope these results contribute to the earlier screening of OA and better clinical treatment strategies in specific populations with neck pain.

## Methods

### Ethics approval

This study was conducted by the guidelines approved by the Research Ethics Review Board of the National Center for Health Statistics (Protocol #2005–6). All patients involved in this study were informed consent from respondents.

### Study design and participants

This study utilized the data from the National Health and Nutrition Examination Survey data set of the USA (NHANES). It was a large cross-sectional study conducted by the National Center for Health Statistics to evaluate the overall health and nutritional status of the population in the USA.

**Fig. 1:**
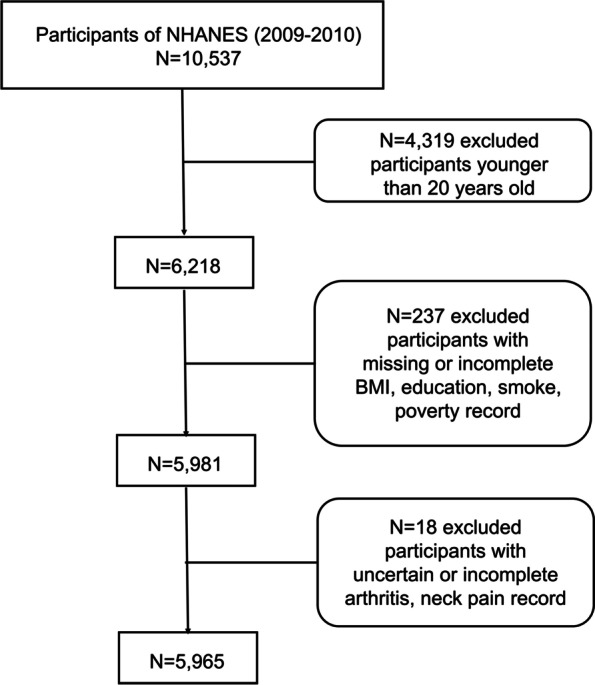
Flowchart of participants in this study

We initially enrolled 10,537 individuals in NHANES 2009–2010. Four thousand three hundred nineteen participants who aged less than 20 years old were excluded. Two hundred thirty-seven participants with missing or incomplete body mass index (BMI), education level information, and smoking records were also ruled out from this study. We have also excluded 17 participants with uncertain or incomplete arthritis and neck pain information. In the end, a total of 5965 participants were included and each participant represented approximately 50,000 individuals (Fig. 1).

### Mortality outcomes determination

NHANES public-use linked mortality file (Dec. 31, 2015) was used to determine the mortality status in the follow-up population through a probability matching algorithm. Disease-specific death was classified by the International Statistical Classification of Diseases, 10th Revision (ICD-10).

### Variable definition

Osteoarthritis status was found in self-reported personal interview data in Medical Conditions Questionnaire (MCQ) During the NHANES interview, participants are asked a series of questions to assess their history of arthritis and joint symptoms. The questions specifically related to OA such as: “Has a doctor or other health professional ever told you that you have arthritis?,” “What type Which type of arthritis was it?”.

Neck pain information was retrieved from the Arthritis Questionnaire Section (ARQ). The investigators will begin by taking a detailed medical history to gather information about the nature of the neck pain. This includes asking questions about the onset of pain, duration, intensity, location, radiation of pain, aggravating and relieving factors, and associated symptoms. They may also inquire about any previous injuries, the age when neck pain started, and when last had neck pain.

This evaluation of osteoarthritis (OA) and neck pain occurs during the physical examination component of the survey. These evaluations occur during the participant's visit to the NHANES Mobile Examination Center (MEC). Chronic neck pain was defined as neck pain that persists for ≥ 1 year.

### Assessment of covariates

Information on participants’ demography and lifestyle factors was acquired from the questionnaires, including age, gender, race/ethnicity, education levels, family income, smoking status, alcohol consumption, and body mass index. Moreover, the race/ethnicity information was categorized as non-Hispanic White, non-Hispanic Black, Mexican American, and other race. The education levels were classified as less than high school, high school or equivalent, and college or above.

### Statistical analyses

All statistical analyses were performed by R software (Version 4.2.2) by CDC guidelines (https://wwwn.cdc.gov/nchs/nhanes/tutorials). All of the estimates were calculated with sample weights to produce representative data of the civilian noninstitutionalized US population. We have established four multivariable logistic regression models between neck pain and osteoarthritis: Crude model, no covariates were adjusted; Model 1: Age, gender, and race were adjusted; Model 2, age, gender, race, education level, and poverty to income ratio were adjusted; Model 3: Age, gender, race, education level, poverty to income ratio, smoke status, body mass index, and bone mineral density were adjusted. In addition, four multivariable Cox regressions were also constructed to figure out the relationship between chronic neck pain and patients’ mortality rate. Crude model, no covariates were adjusted; Model 1: Age, gender, and race were adjusted; Model 2, age, gender, race, education level, and poverty to income ratio were adjusted; Model 3: Age, gender, race, education level, poverty to income ratio, smoke status, body mass index, and bone mineral density were adjusted. Kaplan–Meier and multivariable Cox regression curves were depicted with an R package survey (version 4.0) [20].

## Results

### Baseline characteristics of study participants

In this study, the analysis dataset consisted of data from 5965 participants (mean age 46.91 ± 0.50, 48.21% males). Among them, 8.18% had osteoarthritis and 5.92% suffered from neck pain. The baseline information of the research population divided by osteoarthritis and non-osteoarthritis was presented in Table 1. Compared with the non-osteoarthritis group, the average age and body mass index (BMI) of osteoarthritis patients were significantly higher. The proportion of women was also significantly higher in the OA group than in the non-OA group. There were differences in other demographic data such as race, education level, and smoking status. In addition, the baseline information of the research population classified by neck pain syndrome is shown in Table 2. The total number of 353 patients with neck pain in our study was involved in the analysis. Among them, 181 patients reported experiencing pain for over 1 year, 86 patients reported a duration of pain between one month and 1 year, and another 86 patients reported a duration of less than one month. Furthermore, the average age at which these patients first experienced neck pain was 35.23 (24–66). Participants with neck pain were more likely to suffer from OA. There were also significant differences in sex, race, and smoke status between the neck pain group and the no-neck pain group.

**Table 1:** Characteristics of the participants according to the presence or absence of osteoarthritis

Characteristics	Non-osteoarthritis	Osteoarthritis	*P* value
**Age**	45.53 ± 0.48	61.58 ± 0.58	** < 0.0001**
**BMI**	28.61 ± 0.14	30.35 ± 0.39	**0.001**
**Spine BMD**	1.04 ± 0.00	1.03 ± 0.01	0.31
**Sex**			** < 0.0001**
Female	2785 (50.93)	304 (63.10)	
Male	2692 (49.07)	184 (36.90)	
**Race**			** < 0.0001**
Non-Hispanic White	2519 (66.25)	335 (85.68)	
Non-Hispanic Black	1019 (11.90)	65 (6.47)	
Mexican American	1052 (9.15)	39 (2.51)	
Other race	887 (12.70)	49 (5.33)	
**Education**			0.14
College or above	2624 (57.95)	265 (60.59)	
High school or equivalent	1255 (22.70)	117 (25.15)	
Less than high school	1598 (19.35)	106 (14.27)	
**Smoke**			**0.01**
No	2994 (55.92)	219 (48.78)	
Yes	2483 (44.08)	269 (51.22)	
**Neck pain**			**0.01**
No-Pain	5170 (94.00)	442 (89.03)	
Pain	307 (6.00)	46 (10.97)	

*N* represented the unweighted number, and the *P* value was calculated by weighted values using related NHANES weight

Figures are expressed as mean ± standard error (for age, BMI, and spine BMD). Other figures are expressed with *N* and percent

*BMI* Body mass index, *BMD* Body mineral density

**Table 2:** Characteristics of the participants according to the presence or absence of neck pain

Characteristics	No pain	Pain	*P* value
**Age**	46.96 ± 0.51	46.11 ± 0.72	0.22
**BMI**	28.70 ± 0.13	29.59 ± 0.54	0.13
**Spine BMD**	1.04 ± 0.00	1.05 ± 0.02	0.34
**Sex**			**0.01**
Female	2874 (51.41)	215 (60.21)	
Male	2738 (48.59)	138 (39.79)	
**Race**			**0.01**
Non-Hispanic White	2657 (67.38)	197 (75.74)	
Non-Hispanic Black	1025 (11.53)	59 (9.95)	
Mexican American	1042 (8.79)	49 (5.55)	
Other race	888 (12.30)	48 (8.76)	
**Education**			0.88
College or above	2708 (58.16)	181 (58.47)	
High school or equivalent	1286 (22.85)	86 (23.80)	
Less than high school	1618 (19.00)	86 (17.73)	
**Smoke**			** < 0.0001**
No	3078 (56.22)	135 (42.07)	
Yes	2534 (43.78)	218 (57.93)	
**OA**			**0.01**
Non-osteoarthritis	5170 (91.83)	307 (85.34)	
Osteoarthritis	442 (8.17)	46 (14.66)	

*N* represented the unweighted number, and the *P* value was calculated by weighted values using related NHANES weight

Figures are expressed as mean ± standard error (for age, BMI, and spine BMD). Other figures are expressed with *N* and percent

*BMI* Body mass index, *BMD* Body mineral density

### Associations between neck pain and OA

Based on the analysis of the demographic data, we included all variables in a multivariate logistic regression to confirm the association between neck pain and OA (Table 3). Taking the no-pain population as the reference, neck pain would lead to a higher incidence of OA in both the unadjusted model [1.932 (1.232, 3.028)] and three adjust models (model 1 [2.498 (1.504, 4.146); model 2 [2.600 (1.502, 4.501)] and model 3 [2.519 (1.325, 4.788)].

**Table 3:** Association between neck pain and osteoarthritis

	No Pain	Neck pain (95% CI)	***P* value**
Crude model	ref	1.932 (1.232, 3.028)	0.007
Model 1	ref	2.498 (1.504, 4.146)	0.002
Model 2	ref	2.600 (1.502, 4.501)	0.004
Model 3	ref	2.519 (1.325, 4.788)	0.01

Crude model: no covariates were adjusted

Model 1: age, gender, and race were adjusted

Model 2: age, gender, race, education level, and poverty-to-income ratio were adjusted

Model 3: age, gender, race, education level, poverty-to-income ratio, smoke status, body mass index, and bone mineral density were adjusted

Subgroup analysis also conveyed that neck pain was associated with all subgroup participants including males [3.272 (1.828, 5.859)] and females [2.305 (1.470, 3.614)], smokers [2.522 (1.616, 3.936)] and non-smokers [2.760 (1.519, 5.015)], as well as in most race groups except Mexican Americans (Table 4). These results indicated that the correlation between neck pain and OA is reliable and consistent.

**Table 4:** Subgroup analysis of the association of neck pain and osteoarthritis

	**No pain**	**Pain**	***p***	***p* for interaction**
**Sex**				0.274
Male	ref	3.272 (1.828, 5.859)	** < 0.0001**	
Female	ref	2.305 (1.470, 3.614)	** < 0.001**	
**Race**				0.331
Non-Hispanic White	ref	2.445 (1.556, 3.843)	** < 0.001**	
Non-Hispanic Black	ref	2.517 (1.026, 6.175)	**0.044**	
Mexican American	ref	2.723 (0.748, 9.909)	0.129	
Other race	ref	4.126 (1.565, 10.878)	**0.004**	
**Smoke**				0.875
Yes	ref	2.522 (1.616, 3.936)	** < 0.0001**	
No	ref	2.76 (1.519, 5.015)	** < 0.001**	

All data were adjusted for age, gender (except gender-specific estimates), race (except race-specific estimates), education level, poverty-to-income ratio, smoke status (except smoke-specific estimates), body mass index, and bone mineral density

### Relationships between chronic neck pain with OA patients’ mortality

**Fig. 2:**
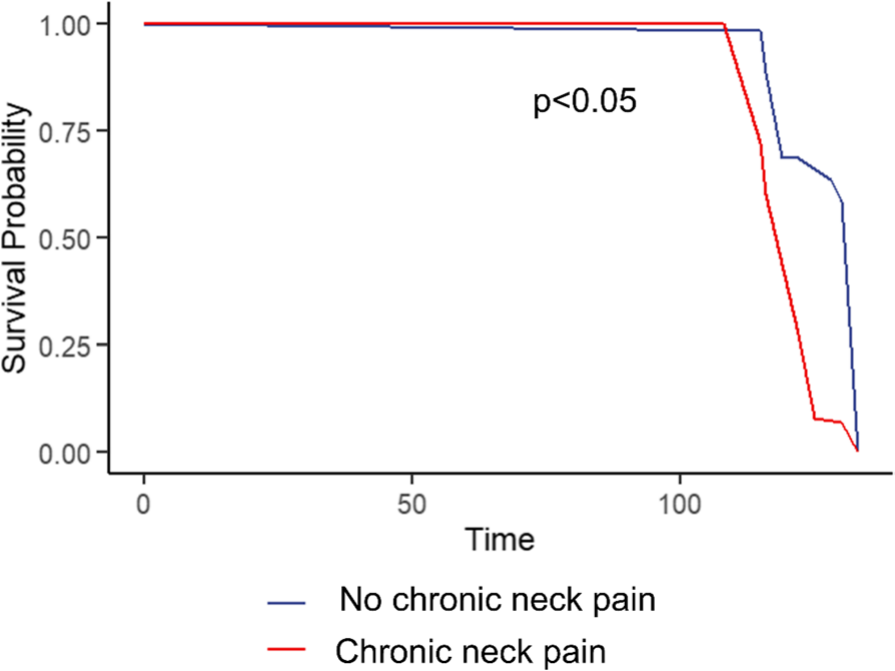
Kaplan–Meier curve of survival probability of OA patients comparing with chronic neck pain and no chronic neck pain. The red line represented the OA patients with chronic neck pain, while the blue line represented the OA patients without chronic neck pain (*p* < 0.05)

To uncover the influence of neck pain on OA patients, we have performed a Kaplan–Meier estimate within follow-up all-cause mortality status information in patients with OA (Fig. 2). Compared with OA patients without chronic neck pain, OA patients who had chronic neck pain (over 1 year) tended to have a significantly lower survival probability (*p* < 0.05). The major cause of death which increased in OA patients with neck pain is chronic lower respiratory diseases.

Four multivariate Cox regression models were also constructed to investigate the independent role of chronic neck pain in OA patients’ mortality (Table 5). All of these models show significantly increased mortality hazard ratios (HRs) in OA patients with chronic neck pain (unadjusted model [2.94 (1.61, 5.37)] adjust model (model 1 [4.74 (1.24, 18.11); model 2 [4.65 (1.88, 45.01)] and model 3 [3.3014 (1.23, 45.85)).

**Table 5:** Association between chronic neck pain and mortality of osteoarthritis patients

	No chronic neck pain	Chronic neck pain (95% CI)	***P* value**
Crude model	ref	2.94 (1.61, 5.37)	0.00047
Model 1	ref	4.74 (1.24, 18.11)	0.022
Model 2	ref	4.65 (1.88, 45.01)	0.025
Model 3	ref	3.3014 (1.23, 45.85)	0.04

Crude model: no covariates were adjusted

Model 1: age, gender, and race were adjusted

Model 2: age, gender, race, education level, and poverty-to-income ratio were adjusted

Model 3: age, gender, race, education level, poverty-to-income ratio, smoke status, body mass index, and bone mineral density were adjusted

**Fig. 3:**
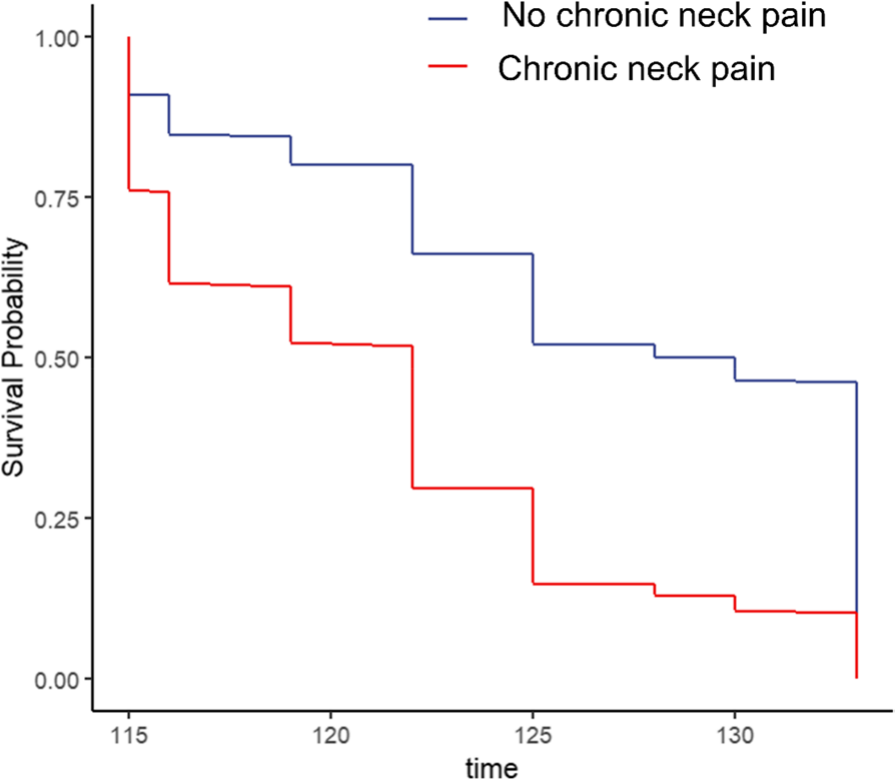
Multivariate adjustment Cox regression plot of survival probability of OA patients comparing with chronic neck pain and no chronic neck pain. The red line represented the OA patients with chronic neck pain, while the blue line represented the OA patients without chronic neck pain. Age, gender, race, education level, poverty to income ratio, smoke status, body mass index, and bone mineral density were adjusted (*p* < 0.05)

The multivariate adjustment Cox regression plot clearly showed the significantly different survival probabilities in OA patients with or without chronic neck pain over 1 year (Fig. 3).

## Discussion

To the best of our knowledge, this is the first prospective study to explore the association between neck pain (NP) and the risk of osteoarthritis (OA). In this cohort of 5965 participants, individuals with a history of neck pain tended to have a positive correlation with osteoarthritis. These associations still remained significant even after accounting for various which potentially related to OA including gender, age, race, education level, family income, smoking status, and BMI. These findings suggest that clinical physicians should screen for osteoarthritis among people with neck pain.

Currently, the underlying mechanism of the association between neck pain and OA remains unclear. Since this study is a cross-sectional investigation, we are unable to establish a causal relationship between the neck pain and OA. Therefore, it remains uncertain whether neck pain increases the risk of developing OA or if individuals with OA are more susceptible to neck pain. The former may be linked to the spinal imbalance resulting from neck pain [2], while the latter could be associated with the systemic inflammatory response of OA [21]. Further research evidence is needed to delve into this conclusion.

Moreover, we have focused on the influence of neck pain on OA patients and found chronic neck pain over 1 year was also positively associated with the all-cause mortality rate in those OA populations. This finding was robust even after adjusting several variations in the proportional hazard models. The major cause of death surged in OA patients with chronic neck pain was chronic lower respiratory diseases.

Previous studies have reported that the mortality risk of OA may vary widely depending on their particular comorbidities [14]. Studies using 633,330 individuals from Spain also illustrated that the most common comorbidity in OA patients was back/neck pain (33.6%), which has significantly higher morbidity compared with other groups (adjusted HR: 1.12 [95% CI: 1.09–1.15]) [17]. Chronic neck pain may limit mobility and physical activity in OA patients, leading to a sedentary lifestyle and reduced overall physical fitness [19]. Physical inactivity is associated with a higher risk of mortality and the development of comorbid conditions [19]. Moreover, functional decline resulting from neck pain can impact activities of daily living, increasing the vulnerability of OA patients to adverse health outcomes [18]. Moreover, the pharmacological management of OA and neck pain often involves the use of analgesics, anti-inflammatory drugs, and other medications [22]. Prolonged use of certain medications, especially opioids and nonsteroidal anti-inflammatory drugs (NSAIDs), can be associated with adverse effects, including gastrointestinal bleeding, renal dysfunction, cardiovascular events, and increased mortality risk [23].

There are some strengths in our study. First, we have utilized a relatively large sample size population to generalize our result based on the nationally representative sample of adults in the USA. Second, these participants have long-term reliable follow-ups, which provide sufficient strength for the analysis in our study. Third, we have managed to improve the effectiveness of our conclusion by adjusting for potential confounding factors including age, gender, race, education level, poverty-to-income ratio, smoke status, body mass index, and bone mineral density.

This study also has several limitations. First, we cannot determine the causality due to this is a cross-sectional study. Prospective cohort studies are still needed to confirm causality later on. Second, the neck pain history and OA diagnosis were based on self-report, and there is no specific location information. Despite this, the NHANES data is considered to be valid for assessing the prevalence of neck pain and OA [24, 25]. The association could have been weakened if undiagnosed OA patients were categorized as healthy controls. We will embark on further longitudinal studies to illustrate the association between chronic neck pain and different locations of osteoarthritis in the future.

## Conclusion

In summary, our study revealed a strong association between neck pain and osteoarthritis. In particular, chronic neck pain over 1 year will increase the mortality of OA patients. These may provide evidence for screening osteoarthritis in neck pain patients and adjusting the treatment strategy promptly.

## Data Availability

The survey data are publicly available on the NHANES website for all researchers worldwide (www.cdc.gov/nchs/nhanes/).

## Abbreviations

BMI: Body mass index
BMD: Bone mineral density
NHANES: National Health and Nutrition Examination Survey
CDC: The Center for Disease Control and Prevention
NCHS: The National Center for Health Statistics
STROBE: Strengthening the Reporting of observational studies in Epidemiology
